# Current Knowledge Regarding the Interaction Between Oral Bone Metabolic Disorders and Diabetes Mellitus

**DOI:** 10.3389/fendo.2020.00536

**Published:** 2020-08-07

**Authors:** Xiaofeng Wang, Huiyu Wang, Tianfu Zhang, Lu Cai, Chenfei Kong, Jinting He

**Affiliations:** ^1^Department of Stomatology, China-Japan Union Hospital of Jilin University, Changchun, China; ^2^Department of Pediatrics, Pediatric Research Institute, The University of Louisville School of Medicine, Louisville, KY, United States; ^3^Departments of Radiation Oncology, Pharmacology, and Toxicology, University of Louisville, Louisville, KY, United States; ^4^Scientific Research Center, China-Japan Union Hospital of Jilin University, Changchun, China; ^5^Department of Neurology, China-Japan Union Hospital of Jilin University, Changchun, China

**Keywords:** diabetes mellitus, periodontitis, peri-implant inflammation, periodontal pathogen, bone metabolism, AGEs, vitamin D

## Abstract

Diabetes mellitus, a major chronic disease affecting human health, has been increasing in prevalence in recent years. Diabetes mellitus can cause bone metabolic disorders in patients, leading to osteoporosis, a higher risk of traumatic fracture, and other bone diseases. Bone metabolic disorders in the oral cavity principally manifest as periodontitis, loss of alveolar bone, and failure of implant osseointegration. In recent years, numerous studies have shown that there is a complex interaction between bone metabolic disorders and diabetes mellitus. This paper reviews the adverse effects of diabetes on oral bone metabolism disorders such as alveolar osteoporosis and bone loss in patients with periodontitis, discusses the potential mechanisms of diabetic bone loss, and suggests potential ways to prevent and treat oral bone loss in patients with diabetes mellitus.

## Introduction

Diabetes mellitus is a metabolic disorder that is characterized by chronic hyperglycemia resulting from insufficient insulin secretion and/or insulin resistance in target tissues (1). The incidence of diabetes is rising rapidly. In 2017, there were 451 million people aged 18–99 with diabetes worldwide (2), and there are expected to be more than 592 million cases by 2035 (3). Poorly managed diabetes is associated with damage to multiple organ systems, including the retinas, nerves, blood vessels, kidneys, and bone (4). Diabetic bone disease is principally a disorder of bone metabolism and of calcium and phosphorus metabolism, which lead to secondary osteopenia, osteoporosis (OP), and other types of diabetic osteopathy. OP is one of the most common chronic complications of diabetes: 50% of diabetic patients have OP (5, 6). OP is a bone metabolic disease characterized by lower bone mass, destruction of bone microstructure, greater bone fragility, and a higher risk of fracture (7). Clinical and *in vitro* cytologic studies have shown that both type 1 (T1DM) and type 2 (T2DM) diabetes mellitus increase the risk of fracture and interfere with bone healing (8). Furthermore, with the aging of the population, OP has become a global problem that affects the health of middle-aged and elderly people.

The systemic bone loss that occurs in diabetes includes alveolar bone resorption and OP. The jaws of diabetic patients often show a loss of alveolar bone and OP, which affects the healing of oral surgical wounds. Furthermore, alveolar bone resorption often occurs in association with denture restoration, periodontal surgery, and the insertion of dental implants, which not only increases the difficulty of prosthetic treatment but also affects the prognosis. The principal manifestations of diabetic alveolar OP are a reduction in alveolar bone mass, damage to the bone microstructure, an increase in the brittleness of the bone, a reduction in the bone density, and a higher risk of fracture (9). It is also closely related to periodontal disease, tooth loosening, tooth loss, other oral diseases, and the placement of implants (10). Oral bone maybe one of the earliest sites from which bone loss occurs. More specifically, the jaw may manifest OP first, followed by reabsorption of alveolar bone (11). It has been shown that hyperglycemia inhibits new bone formation and exacerbates alveolar bone resorption due to periodontal disease, which is a frequent cause of implant failure (12). Abnormal bone metabolism or peri-implant inflammation, which develops secondary to hyperglycemia, can result in serious alveolar bone defects in the region of the implant and ultimately lead to a failure of osseointegration, making diabetes a relative contraindication for implant repair (13). However, the relationship between diabetes and oral bone loss is not fully understood.

Here, we review the characteristics of systemic bone metabolic disorder caused by diabetes, and more specifically we review the relationship between diabetes and oral bone loss.

## Review Criteria

The following words were used as search terms to search for English articles in PubMed: “diabetes,” “periodontal disease,” “periodontitis,” “*P*. *gingivalis*,” “infection,” “gingiva treatment,” “clinical trial,” “systematic review,” “meta-analysis,” “mechanism,” and “pathogenesis.” The main focus was articles published during the past 10 years. Reference lists of the identified articles were also reviewed. Endnote X9 was used for electronic management of the literature.

## Study Inclusion and Exclusion Criteria

When collecting the literature, the title and abstract of the literature were selected according to the following inclusion criteria: English, retrospective and prospective clinical trials, observation studies, cross-sectional studies, cohort studies and case series. During this process, selected publications were evaluated according to the following exclusion criteria: case reports with fewer than 10 patients and papers published within the last 10 years.

## Clinical and Epidemiologic Characteristics of Oral Bone Metabolic Disorders Caused by Diabetes Mellitus

Periodontitis is one of the common diseases encountered in stomatology and is slowly progressive. The progression of inflammation can cause the destruction of connective tissue and alveolar bone reabsorption, which ultimately leads to tooth loss. Indeed, it is the principal cause of adult tooth loss (14–16). In addition to affecting oral function, periodontitis has also been shown to be closely associated with cardiovascular and cerebrovascular disease, diabetes, tumors, and other systemic diseases (17). The relationship between diabetes and periodontitis is bidirectional, with each conferring a higher risk of the other (18, 19). Epidemiologic studies have confirmed that diabetes is an important risk factor for periodontitis, and it has been recognized that periodontitis is the sixth-most frequent complication of diabetes (20). The incidence rate of periodontitis in diabetics is obviously higher. Compared with those with normal blood glucose, the characteristics of periodontitis are more severe, periodontal tissue is obviously damaged, and the disease develops rapidly. The clinical manifestations are gingival swelling and bleeding, root exposure and bifurcation lesions in severe cases, and recurrent periodontal abscesses, which eventually lead to tooth loosening and loss (21, 22). Diabetic patients with poorly controlled blood glucose are at a higher risk of tooth loss, which is more significant in people aged 18–44 (23). Periodontitis is also a potential risk factor for T2DM because it interferes with glucose homeostasis, inducing a pre-diabetic state. It also increases the risk of frank diabetes and its associated complications, including the risk of mortality. The presence of periodontal sites with a clinical loss of attachment of more than 3 mm increases the risks of both diabetes and of mortality by 1% (24).

A few studies have determined the clinical and epidemiologic characteristics of the disordered oral bone metabolism associated with diabetes (Table 1). A survey of 4,477 adults aged ≥ 30 showed that 43.7% of the people with diabetes had periodontitis, while only 25.0% of the people without diabetes had periodontitis. In this study, smoking, oral pain, and failure to use oral cleaning products were risk factors for periodontitis in the diabetic patients (29). In another study that compared 60 patients with T2DM and 40 healthy people, the salivary glucose concentration of diabetic patients was found to be significantly higher than that of healthy people, and high salivary glucose can significantly affect the periodontal status of patients with T2DM (26).

**Table 1:** Clinical and epidemiologic characteristics of the oral bone metabolic dysregulation associated with diabetes.

**Authors**	**Types of diabetes**	**Number of cases**	**Principal findings**
Meenawat et al. (25)	T1DM	28	The severity of gingivitis and periodontitis in T1DM patients is significantly higher than that in healthy people.
Puttaswamy et al. (26)	T2DM	T2DM: 60; Healthy people: 40	The salivary glucose concentration in diabetic patients is significantly higher than that in healthy people, and periodontal status is poorer.
Liljestrand et al. (27)	T2DM	8,446	The risk of T2DM in patients with moderate-to-severe periodontitis is 3.2 times higher than that in patients with mild or moderate periodontitis.
Al Zahrani et al. (28)	T2DM	Well-controlled: 35; Poorly controlled: 32	Patients with poorly controlled T2DM demonstrate worse peri-implant bone outcomes than patients with well-controlled T2DM.
Hong et al. (29)	T1DM and T2DM	4,473	43.7% of people with diabetes have periodontitis compared with only 25.0% of the general population.
Annibali et al. (30)	T1DM and T2DM	Implants: 1,142	Patients with diabetes are more likely to suffer implant failure during the period of osseointegration and the first year of loading.

T2DM accounts for about 90% of the clinical cases of diabetes (31) and, because of the pathologic changes caused by T2DM, the incidences of periodontitis and tooth loss increase. T2DM may increase the host's inflammatory response to oral flora, which may in turn increase the risk of periodontitis and gingivitis in susceptible populations (32). Several prospective studies have shown that the risk of developing T2DM in patients with moderate-to-severe periodontitis is 3.2 times higher than that in patients with mild or moderate periodontitis (27, 33). However, the relationship between T1DM and periodontitis requires further exploration. The severity of gingivitis and periodontitis in T1DM patients is significantly higher than that in healthy people (25), but there is insufficient evidence to suggest that periodontal inflammation in T1DM patients is associated with poor glycemic control (34).

The development of bone defects after implantation is a manifestation of peri-implant inflammation. Similar to periodontitis, peri-implant inflammation is a destructive disease of the periodontal tissue that is regulated both by plaque initiation and host factors. Diabetes mellitus is also a high risk factor for peri-implant inflammation. An increasing number of studies have demonstrated a relationship between diabetes mellitus and peri-implant inflammation (35). Kolklevold et al. (36) have found that diabetic patients have a much higher risk of peri-implant inflammation than other patients, and a clinical study conducted in diabetic patients has shown that the risk of implant failure in diabetic patients is 4.8% higher than in other patients (37). Furthermore, a retrospective study has shown that the success rate of implantation in diabetic patients with good blood glucose control is significantly higher than that in patients with equivocal or poor control (38, 39). Other studies have shown that successful implant osseointegration is less likely in diabetic patients than in healthy people, and that this is often accompanied by incomplete or delayed bone healing and the formation of immature bone (40). It has also been shown that there is a progressive increase in the incidence of implant failure during the period of osseointegration and the first year of loading in diabetic patients (30). A meta-analysis has shown that the incidence of marginal implant bone defects is higher in diabetic patients (13, 41), and systematic retrospective studies have indicated that patients with poorer glycemic control show more severe bone resorption at the implant margins (28). However, patient periodontal history has a stronger relationship with peri-implant inflammation than with diabetes (42), and the relationship between periodontal disease and peri-implant inflammation requires further clinical and laboratory investigation (43).

## Effects of Diabetes on Oral Bone Metabolism

### Potential Mechanisms

Bone metabolism is a dynamic process, facilitating bone resorption and reconstruction between embryonic development and the end of life. Bone remodeling involves a balance between the actions of osteoblasts and osteoclasts, and when there is an imbalance in their activities, bone metabolic disorders manifest (44). Bone metabolism involves two basic processes: bone formation and resorption (45). Osteoclasts are derived from hematopoietic stem or precursor cells and their principal function is to induce bone resorption (46). Osteoclast-regulated bone resorption begins when osteoblasts initiate the proliferation of osteoclast precursors and promote the differentiation of these precursors into mature osteoclasts by the secretion of macrophage colony-stimulating factor (MCSF). MCSF not only reduces the apoptosis of osteoclasts and prolongs the cell cycle, but it also promotes the proliferation, differentiation, and maturation of osteoclasts (47). Nuclear transcription factor (NF-κB) receptor is required for osteoclast formation and is regulated by osteoprotegerin (OPG) feedback. Diabetes affects osteoclasts and osteoblasts in the periodontal membrane in different ways, resulting in an unbalanced relationship between bone absorption and repair. Some studies have focused on the roles of factors affecting osteoclast generation during periodontal infection in diabetic patients, such as receptor activator of nuclear factor B ligand (RANKL) and osteoprotegerin (OPG). RANKL is a ligand of RANK and is mainly expressed in osteoblasts, fibroblasts and activated T or B lymphocytes, or is secreted into the extracellular matrix. RANK on the surface of preosteoclast cells recognizes and binds to RANKL, resulting in the differentiation into mature osteoclasts. OPG is another receptor of RANKL that inhibits osteoclast development. Glycemic control affects the expression of RANKL and OPG, and the proportion of RANKL and RANKL/OPG in gingival crevicular fluid of diabetic patients with poor glycemic control is higher than that of diabetic patients with good glycemic control (48). However, glucose control was reported to have no effect on the expression of RANKL mRNA in periodontal tissue. Antonoglou et al. (49) reported the serum levels of RANKL and OPG in patients with periodontitis and type 1 diabetes, and found that the severity of periodontitis correlated with the serum level of OPG. Zhang l et al. evaluated changes in the RANKL/OPG ratio before and after periodontal intervention in patients with chronic periodontitis (CP) and type 2 diabetes mellitus (T2DM). They found that after periodontal intervention, both the well-controlled and poorly controlled subgroups exhibited significant increases in OPG and decreases in RANKL in serum, and the R/O ratio was also notably reduced (50). These studies partly explain the severity of alveolar bone resorption in diabetic patients.

**Figure 1:**
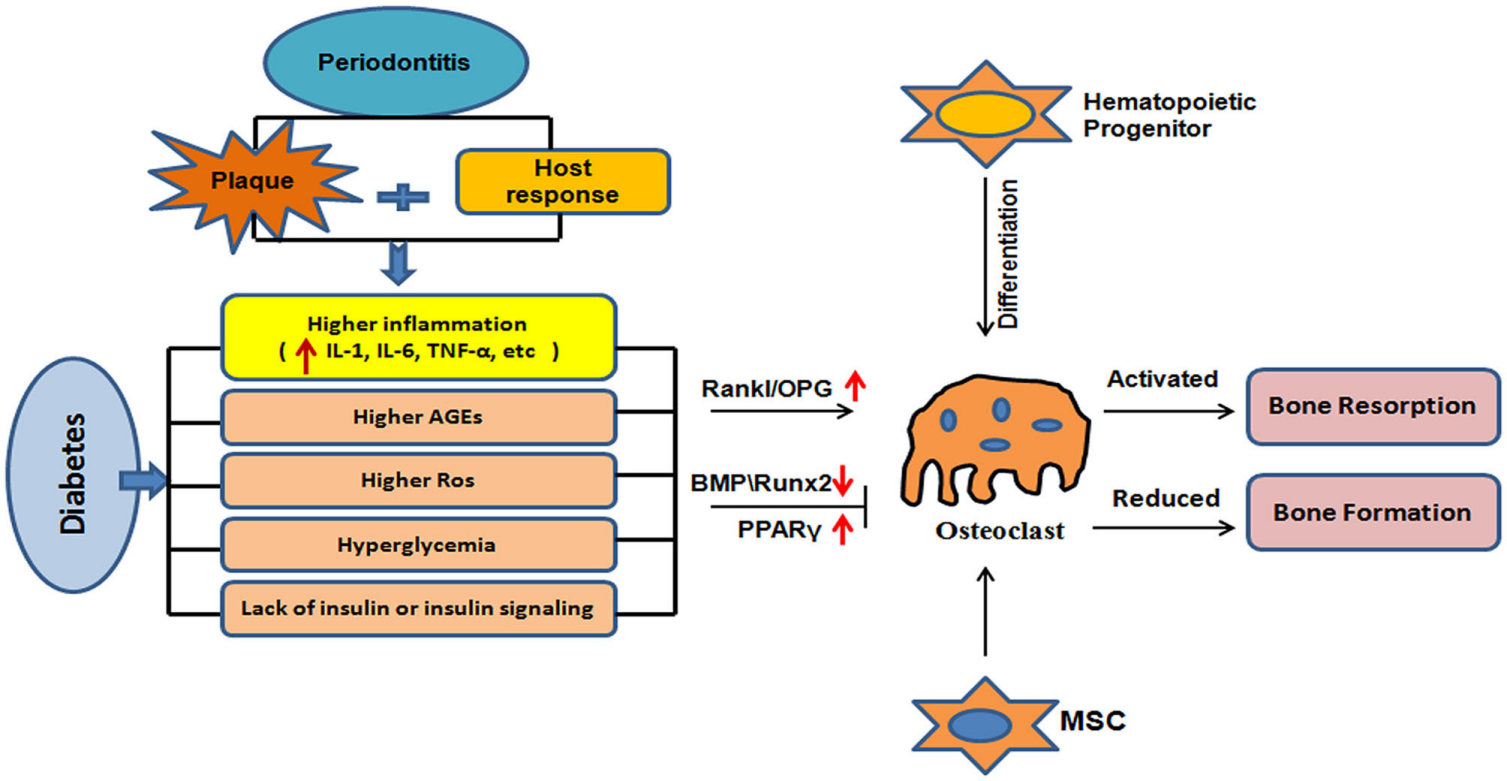
Mechanisms of bone metabolic disorder induced by diabetes. Diabetes leads to hyperglycemia because of a lack of insulin or insulin resistance; increases inflammation; and results in the generation of advanced glycation end-products and reactive oxygen species. This dysregulation and an increase in the RANKL/OPG ratio may lead to greater osteoclast production, thereby promoting bone resorption. Diabetes is associated with hyperglycemia, inflammation, and the generation of AGEs and ROS. This may lead to a reduction in the expression of BMPs and Runx2, and an increase in the expression of PPARγ and/or other mechanisms that reduce bone formation.

Osteoblasts are derived from mesenchymal stem cells (MSCs) and participate in bone formation. Type I collagen secreted by osteoblasts forms the basis of the organic bone-like matrix, which mineralizes, along with alkaline phosphatase, osteocalcin, and osteopontin, ultimately forming mature hydroxyapatite crystals (51). Alkaline phosphatase is a phenotypic marker of osteoblasts that directly reflects the activity of these cells. Mukaiyama et al. (52) tracked 626 postmenopausal women with OP and confirmed that the high serum alkaline phosphatase in OP patients is the result of high bone turnover. The expression of alkaline phosphatase, osteocalcin, and osteopontin is upregulated during osteogenesis, and they are therefore considered markers of the differentiation and maturation of osteoblasts. However, diabetes is associated with reductions in alkaline phosphatase, osteocalcin, and osteopontin expression (53, 54). In addition, bone morphogenetic protein (BMP) is an important regulator of osteogenesis because it induces MSCs to differentiate into osteoblasts (55), and it regulates osteoblast activity (56) *via* the Smad and p38-mitogen-activated protein kinase (MAPK) signaling pathways. It achieves these effects by binding to its receptor (BMPR) and phosphorylating it, and the activated BMPR activates R-Smad in turn, to promote osteogenesis and the differentiation of stem cells (57). In addition, the BMP–BMPR complex can act on transforming growth factor kinase 1 (TAK1), through osteopontin, to activate the p38-MAPK signaling pathway and increase the expression of osteocalcin, osteoside, and other factors, which accelerates bone formation (58). However, hyperglycemia is associated with a reduction in the expression of BMP, an inhibition of the differentiation of MSCs into osteoblasts, and a reduction in bone formation (59). Hyperglycemia and insulin deficiency reduce the expression of runt-related transcription factor-2 (Runx2) and inhibit bone formation (60, 61). In addition, hyperglycemia promotes adipogenesis from MSCs by activating peroxidase proliferator-activated receptor-gamma (PPARγ), which downregulates bone formation and thus bone mass (62). The mechanisms of bone metabolism that are affected by diabetes are summarized in Figure 1.

### Periodontal Pathogen

Oral flora is an important contributor to complications of diabetes. In the pathogenesis of periodontitis, plaque formation and autoimmunity lead to the differentiation and proliferation of a large number of osteoclasts, increasing bone resorption above the rate of bone formation, which leads to net alveolar bone resorption (63, 64). An imbalance in normal oral flora or a decline in immunity can lead to an imbalance between periodontal microorganisms and the host, and aggravation of the inflammatory response can lead to periodontal disease, which further leads to an imbalance in oral bone metabolism. “Red complex” gram-negative bacteria, which are mainly composed of *Tannerella forsythia, Porphyromonas gingivalis*, and *Treponema*, are considered to be the main pathogenic microorganism of periodontitis (65). Early studies showed that the detection rate and levels of periodontal pathogens were different between patients with type 2 diabetes periodontitis and those without periodontitis. However, recent studies found that periodontal disease under the gum is associated with plaque containing *A. actinomycetes, F. nucleatum, P. intermedia*, and other microorganisms in diabetic patients and there was a statistically significant difference between healthy people (66), suggesting that diabetes may change host immune cells and cytokine levels and lead to periodontal balance between microorganisms and the host, the body's inflammatory response is aggravating, lead to periodontal disease. Graves DT et al. showed that diabetes enhanced inflammatory cytokine IL-17 levels in diabetes alters the oral microbiome, making it more pathogenic. Treatment of oral mucosa with an IL-17 antibody decreased neutrophil recruitment induced by diabetic oral flora, as well as IL-6 and RANKL and bone resorption (67).

### Immuno-Inflammatory Responses

Several studies have shown that monocytes in diabetic patients have a higher inflammatory phenotype than those in healthy people, resulting in a systemic inflammatory response. The functions of inflammatory cells, such as neutrophils, monocytes, and macrophages, are altered in diabetic patients. These cells produce high levels of interleukin (IL) 1, tumor necross factor-cytokines (TNF-), and prostaglandin E2 when stimulated by the endotoxins of periodontal pathogens. This in turn increases cytokines and inflammatory mediators in gingival crevicular fluid. Impaired adhesion, chemotaxis and phagocytosis of neutrophils in diabetic patients are likely to increase periodontal pathogen survival in periodontal pockets (68). Studies have shown that T lymphocytes clustered in insulin-sensitive tissues may influence glucose metabolism by regulating macrophage function. Duarte et al. (69) proposed that the T lymphocytes clustered in periodontal tissues of patients with periodontitis with diabetes were mainly helper T cells 17 and regulatory T cells, suggesting that such T lymphocytes might be related to the development of periodontal disease in diabetic patients. At the same time, higher levels of IL-17 were detected in gingival crevicular fluid in these patients, and the increase of IL-17 was more significant in diabetic patients with poor blood glucose control, further demonstrating the role of helper T cell 17 in the development of periodontal disease in diabetic patients. Increased interactions between macrophages and monocytes in diabetic patients leads to increased secretion of pro-inflammatory cytokines such as IL−1, IL−6, and TNF-, which may eventually activate osteoclasts and collagenase/matrix metalloproteinases, leading to alveolar bone destruction. Silva et al. found that in an experimental model of type 1 diabetic periodontal disease, IL−23, IL−17, Mmp8, and Mmp14 expression were significantly increased while IL−6 and IL−10 expression were decreased, suggesting a decrease in the response of DM1 to Th1. The increase in IL−17 expression suggested activation of the Th17 pathway, which led to a large recruitment of neutrophils (70).

Good periodontal care can reduce the serum glycated hemoglobin A1c (HbA1c) concentration by ~0.4%, which implies better blood glucose control, in addition to the prevention of alveolar bone resorption. Therefore, it is thought that periodontal health should be promoted as an important part of diabetes management (71). A potential mechanism of diabetes-related alveolar bone loss in the presence of periodontitis is presented in Figure 1. Diabetes influences the immune response to oral bacteria and increases local infiltration with pro-inflammatory cells (72). In the diabetic state, the accumulation of AGEs in periodontal tissue increases this inflammatory infiltration and ROS production, resulting in the destruction of bone tissue (73). *In vitro* studies (74) have confirmed that TNF-α inhibits the proliferation and differentiation of osteoblasts, promotes the apoptosis of osteoblasts, and activates osteoclasts, effects that are very similar to the proposed mechanism of the diabetes-associated bone defects. Chronic hyperglycemia accelerates the accumulation of AGEs, and the binding of AGEs to RAGEs can induce osteoblast apoptosis through the MAPK pathway and regulate osteoclast bone resorption (75). Ko et al. (76) have shown that the upregulation of TNF-α in diabetic mice may inhibit new bone formation, and another study (73) has shown that the IL-6 concentration in gingival crevicular fluid is significantly higher in patients with T2DM and periodontitis than in those with simple periodontitis. IL-6 and other pro-inflammatory factors can stimulate osteoclast differentiation and bone resorption, and inhibit bone formation, resulting in bone defects.

### Diabetes and Dental Implants

DM is associated with poorer osseointegration of dental implants, mainly due to microangiopathy, which slows down wound healing and impairs the immune response of periodontal tissues to infection. A high glucose concentration can also directly inhibit the proliferation of osteoblasts and collagen production, and stimulate bone resorption. In addition, it is not conducive to the formation of bone matrix, negatively affecting the adhesion, expansion, and accumulation of extracellular matrix (13, 77). The accompanying increase in AGEs promotes the release of inflammatory cytokines, such as IL-1, IL-6, and TNF-α, further increasing the activity of osteoclasts, leading to a deterioration in bone quality and affecting osseointegration (30). Al-Sowygh et al. (28) have confirmed that the bone defects surrounding implants are worse in individuals with hyperglycemia and high AGE concentrations. In addition, T1DM is more harmful than T2DM in this regard and is associated with a higher failure rate of implant osseointegration. At present, the importance of the duration of diabetes for implant osseointegration and the specific mechanism involved remain to be determined. Most previous reports have not provided data regarding the timing of the diagnosis of diabetes, and whereas some researchers have suggested that the duration of diabetes may influence implant failure, others do not believe that this is a major factor (78). Nevertheless, it is generally thought that good glycemic control is necessary for successful osseointegration in patients with diabetes (79).

## Hyperglycemia, AGEs, and ROS

In the presence of sustained hyperglycemia, sugars combine with proteins or lipids to form AGE. Receptors for AGE (RAGE) are found in a wide variety of cells. Hyperglycemia leads to increases in both AGE and RAGE concentrations in many tissues, including bone matrix. The interaction of AGE-RAGE is closely related to the aggravation of various inflammatory reactions and is involved in the process of collagen and bone metabolism, which may be an important mechanism underlying the increase in the occurrence of periodontitis in diabetes patients. Previous studies have shown that the expression of AGEs is higher in the gingival tissues of patients with diabetic periodontitis (80, 81). When AGEs accumulate in tissues, they activate the NF-κB pathway, which initiates inflammation, promotes the production of matrix metalloproteinase, IL-6, and IL-8 by gingival fibroblasts, and increases collagen degradation in periodontal tissue and alveolar bone resorption (69, 82, 83). Other studies have demonstrated that RAGE is expressed on the surface of specific cells and upregulated in hyperglycemia, increasing the sensitivity of the cells to AGEs (84). When AGEs accumulate in the extracellular matrix, they bind to RAGE on the surface of osteocytes, which increases the generation of intracellular ROS and inflammation, which involves the activation of a series of signal transduction pathways, including p38 MAPK, c-Jun N-terminal kinase, Rho GTPase, PI3K (Phosphoinositide 3-kinase), JAK/STAT, and NF-κB (85). Recent studies have also shown that AGEs can promote inflammation, increase the expression of pro-inflammatory cytokines, and affect the differentiation and function of osteoblasts. In addition, they upregulate osteoclast activity and production, promoting bone resorption, and ultimately form diabetic osteopathy (6). In a mouse model of oral infection with *P. gingivalis*, increased alveolar bone loss in diabetic animals was associated with higher expression of RAGE, inflammatory AGEs, and tissue destruction matrix metalloproteinases than in non-diabetic controls (50). These findings suggest that AGE-RAGE interactions lead to an excessive inflammatory response to bacteria, resulting in tissue destruction in patients with diabetic periodontitis.

In addition, the high concentration of AGEs in diabetic patients causes increases the interaction between macrophages and monocytes, leading to greater secretion of pro-inflammatory cytokines, such as interleukin (IL)-1, IL-6, and TNF-α. TNF-α may eventually activate osteoclasts and collagenase/matrix metalloproteinases, leading to alveolar bone destruction. Furthermore, the production of AGEs is also regulated by ROS: an increase in ROS can further increase AGE concentrations, and ROS also plays a direct role in periodontal tissue destruction. Other cells, such as fibroblasts, may also increase matrix metalloproteinase production and decrease collagen levels in the presence of high concentrations of AGEs. This series of events may lead to an aggravation of the response of periodontal tissue to local pathogens, accelerating the destruction of connective tissue and alveolar bone resorption in diabetic patients. IL-6 and TNF-α are the main inducers of acute phase proteins, including C-reactive protein, and studies have shown that they can disrupt intracellular insulin signaling, leading to insulin resistance and worsening of glucose control in diabetic patients (86, 87).

Recently, Patil et al. (88) have shown that oxidative stress levels are higher in patients with T2DM periodontitis, and the depth of exploration is positively correlated with the serum ROS concentration. ROS concentrations are closely associated with abnormal bone metabolism, possibly because ROS can inhibit the differentiation of bone marrow MSCs into osteoblasts (89). Some previous studies (90) have shown that the concentration of ROS in the gingival crevicular fluid of patients with T2DM and periodontitis is significantly higher than that of non-diabetic periodontitis patients and healthy individuals. Farr et al. (91) have described the mechanism underlying the effects of AGEs on bone tissue. The accumulation of AGEs on bone collagen is considered to be the key component of the pathogenesis of diabetic osteopathy. In diabetes, blood glucose, intracellular ROS concentrations, and carbonyl stress in circulating cells are higher, which together induce the excessive production of AGEs. When AGEs bind to collagen in the extracellular matrix of bone, they directly impair the mechanical properties of bone, including its strength and ability to yield. In addition, the collagen binding of AGEs and their interaction with RAGE in bone cells inhibit the function of bone cells, reducing bone metabolism and eventually leading to diabetic osteopathy. The increase in AGEs, such as pentosan, in bone matrix leads to a gradual increase in the brittleness of bone collagen (92). Previous studies have shown that an increase in urine pentosan concentration is associated with a higher risk of fracture in T2DM (93), and urinary pentosidine levels negatively associate with trabecular bone scores in patients with type 2 diabetes mellitus (94). Brittle bone matrix reduces the ability of bone tissue to resist micro-damage; therefore, the negative effect of diabetes on the ability of bone to resist fracture is not solely due to a reduction in bone strength but also to greater brittleness. It is worth noting that both *in vivo* and *in vitro* studies have shown that the use of a combination of anti-diabetic drugs and anti-OP drugs can ameliorate the effects of AGEs on osteocytes (95, 96), implying that this is a good choice for the treatment of diabetic osteopathy.

## Effects of Calcium, Phosphorus, and Vitamin D Imbalances on Bone Metabolism, Diabetes, and Periodontitis

Abnormal bone metabolism is the principal cause of bone destruction and OP, and calcium and phosphorus are the main components of bone tissue. The excretion of calcium and phosphorus is significantly greater in OP patients. Furthermore, a large number of studies have shown that renal defects can cause parathyroid hyperfunction and disorders of calcium/phosphorus metabolism, leading to lower bone mineral density (BMD), abnormal bone metabolism, and a higher risk of OP. Hyperglycemia has a direct toxic effect on osteoblasts, inhibiting their differentiation. It also has a negative impact on the production of bone matrix and its components (97, 98). However, the osmotic diuresis caused by hyperglycemia also results in a substantial loss of calcium, magnesium, and phosphorus from the body, resulting in lower circulating concentrations and mineral imbalances (99). When the serum concentrations of calcium and phosphorus decrease, the feedback regulation of PTH is disrupted, which promotes the release of calcium from bone into the blood, resulting in further loss of calcium (100). In addition, DM is associated with higher concentrations of many inflammatory factors, including interleukins, cyclooxygenase, prostaglandins, and TNF-α, which can upregulate osteoclast differentiation and induce bone resorption (101, 102). Therefore, to repair jaw defects in diabetic patients, we should first improve glycemic control to help reduce bone resorption.

Vitamin D is an essential nutrient that is required for the maintenance of bone health and thus normal bodily activity (103). Vitamin D itself is not biologically active and must be converted into its active form, 1,25-dihydroxyvitamin D3 (1,25 (OH) 2D3) (104), to have effects. 1,25 (OH) 2D3 is a regulator of both osteoblast-mediated bone formation and osteoclast-mediated bone resorption, and therefore a deficiency affects bone formation (105). Vitamin D achieves its effects by regulating the metabolism of calcium and phosphorus, and it is often used to treat calcium and phosphorus imbalances in OP patients (106). Furthermore, it contributes to calcium absorption and deposition in bone (107, 108). In diabetic patients, who have a higher risk of fracture, this risk is reduced by increasing the serum vitamin D concentration (109). In addition, adequate vitamin D and calcium supplementation can slow bone resorption and accelerate bone formation (110).

Calcium and vitamin D deficiency are also major risk factors for periodontal disease. Previous studies have found that dietary intake of nutrients such as calcium and vitamin D is associated with periodontal health. Lee et al. (111) have shown that the prevalence of periodontal disease in Korean adults is inversely proportional to consumption of dairy products. With respect to oral bone metabolism, vitamin D deficiency not only reduces jaw BMD but also affects the incidence and progression of chronic inflammatory diseases, such as periodontitis (112). A repeated-measures cross-sectional study assessed associations between total vitamin D intake and periodontal health in older men. Total vitamin D intake ≥800 iu was associated with severe periodontal disease (OR = 0.67, 95% ci = 0.55–0.81), while moderate to severe ABL (OR = 0.54, 95% ci = 0.30–0.96) was associated with intake of <400 iu/D. Some studies have concluded that taking vitamin D may prevent the progression of periodontal disease (113). Menzel et al. (114) have analyzed the relationship between vitamin D in the gingival epithelium and loss of alveolar bone, and suggested that dietary restriction of vitamin D leads to alveolar bone loss and increased inflammation in the gingiva. Boggess et al. (115) have found that vitamin D and calcium supplementation increase jaw BMD and inhibit alveolar bone loss, and Garcia et al. (116) have reported that supplementation with 800–1,000 IU vitamin D per day reduces the severity of periodontal disease.

It has also been reported that vitamin D status may be associated with many chronic diseases, including diabetes, cancer, and autoimmune diseases (117–119). In particular, high serum vitamin D concentrations are associated with a lower incidence of diabetes (120). This may be explained by the participation of 1,25 (OH) 2D3 in the regulation of glucose tolerance, achieved through the stimulation of insulin secretion and the enhancement of insulin sensitivity. 1,25 (OH) 2D3 has been shown to maintain glucose homeostasis and promote bone remodeling in diabetic rats, thus ameliorating the adverse effects of diabetes on bone integration (121). A recent *in vitro* study showed that 25-hydroxyvitamin D3 has a positive regulatory effect on periodontal inflammation in diabetic mice via the SOCS3/STAT signaling pathway (122). Over the past 30 years, mounting evidence has suggested a role for vitamin D in glucose homeostasis (123, 124). A previous meta-analysis has shown a relationship between low serum vitamin D and the incidence of T2DM or metabolic syndrome (123), and Al-Timimi et al. (125) have shown that more than two-thirds of T2DM patients are vitamin D deficient, and this deficiency is worse in patients with poor long-term glycemic control. However, other studies have found no relationship between vitamin D and blood glucose (126). Furthermore, vitamin D deficiency is very common in the general population, which hampers the interpretation of any relationship between fracture risk and vitamin D deficiency in diabetic patients (127).

## Prevention and Treatment of Oral Bone Loss in Patients With Diabetes Mellitus

The reduction of alveolar bone loss in diabetic patients with periodontitis and the improvement of the success rate of dental implant bonding mainly depend on effective periodontal treatment, the maintenance of oral hygiene, appropriate anti-infection measures, the maintenance of euglycemia, and the administration of drugs to promote bone healing.

### Basic Periodontal Therapy

Basic periodontal therapy is the first-line treatment for diabetic periodontitis. However, aberrant glucose metabolism is associated with a higher expression of inflammatory mediators and promotes bone resorption, which results in greater susceptibility to periodontal inflammation and accelerates alveolar bone destruction. Therefore, basic periodontal treatment for diabetic periodontitis should be accompanied by appropriate glycemic control. Previous studies have shown that subgingival scaling and root planning to remove subgingival plaque can also be used to effectively treat chronic periodontitis in patients with diabetes mellitus (128). The severity of periodontitis and hyperglycemia are closely related, and if periodontal inflammation can be relieved, this may also ameliorate insulin resistance by promoting insulin receptor function (129). Furthermore, fasting blood glucose and HbA1c concentrations are significantly improved in patients with chronic periodontitis and T2DM after ultrasonic scaling and root surface leveling (130). Thus, the important role of basic periodontal treatment in diabetic patients cannot be ignored. Indeed, another study (131) confirmed that good periodontal treatment and oral hygiene can help glycemic control, reduce inflammation around implants, and reduce bone loss. Treatment of periodontitis in diabetic patients has been shown to improve glycemic control with a 3–4 mmol/mol (0.3–0.4%) reduction in glycosylated hemoglobin in the short term (3–4 months) after treatment. Dental teams have an important role to play in the management of patients with diabetes, given that treatment of periodontitis can lead to clinically relevant hemoglobin a1c reductions (132).

### Systemic Glucose Management Therapy

As described above, hyperglycemia promotes the development of bone defects, such that there is an inverse relationship between glycemic control and the severity of bone defects surrounding implants. Thus, effective management of blood glucose before and after the insertion of an implant is very important for the prevention of bone loss and for optimal bone integration. A previous animal study (31) has shown that insulin therapy can improve bone formation around implants, increase bone-to-implant contact, and promote bone integration. In addition, other hypoglycemic drugs, such as aminoguanidine, metformin, and voglibose, are thought to promote osseointegration (133). Metformin has been shown to significantly inhibit local inflammation induced by periodontal pathogens, reduce cytokine concentrations in gingival crevicular fluid, and improve the clinical index of periodontal disease in patients with diabetic periodontitis (134). Therefore, metformin has dual efficacy in the treatment of diabetic periodontitis. At present, chitosan-based sustained-release metformin preparations for the treatment of chronic periodontitis are undergoing clinical trials (135).

### Systemic or Local Adjuvant Therapy

Although the administration of antibiotics to diabetics is controversial due to their abnormal immune system, diabetics are more susceptible to local bacterial infection and local inflammatory infiltration than healthy people. Therefore, careful consideration is still recommended before administering antibiotics. Anaerobic and facultative anaerobes are the main pathogens causing periodontal infection, and the local application of nitroimidazole drugs has a therapeutic effect in chronic periodontitis (136). Previous studies have reported that supportive treatment with prophylactic antibiotics and mouthwash improves implant survival and reduces postoperative complications (78).

In recent years, the use of vitamin D3 supplementation for patients with diabetic periodontitis has been investigated because of the anti-inflammatory and immunomodulatory effects of this vitamin. Vitamin D3 effectively alleviates periodontal connective tissue damage and bone resorption caused by plaque stimulation and poor glycemic control by inhibiting the expression of pro-inflammatory factors, enhancing the defensive capacity of gingival epithelium, and promoting osteoblast function. In addition to ameliorating defects in bone metabolism, vitamin D3 may also ameliorate insulin resistance (137), making it potentially of great clinical value. Significantly, Wu et al. (138) found that the concurrent administration of insulin and vitamin D3 effectively improves the osseointegration of implants in diabetic mice. Some beneficial effects have also been noted when intra-oral drugs have been used to promote the osseointegration of implants in diabetic patients. Because of the greater local secretion of pro-inflammatory factors and the accelerated bone resorption present in DM patients, research has focused on the local application of anti-inflammatory drugs. Specifically, Wang et al. (139) have shown that a controlled-release anti-TNF-α antibody system reduces the expression of local pro-inflammatory factors and promotes bone healing in diabetic mice.

## Conclusion and Perspectives

As living standards have improved, many people's lifestyles have changed, and many lifestyle-related diseases, such as diabetes and OP, have been increasing in prevalence. Currently, the two-way relationship between diabetes and periodontitis is well-established. Diabetes mellitus is a major risk factor for periodontitis, and the severity of periodontitis seems to influence glycemic control and the development of complications in patients with diabetes mellitus. Future studies should examine the interaction between the two diseases in large clinical trials.

This paper reviews the adverse effects of diabetes on oral bone metabolism disorders such as alveolar osteoporosis and bone loss in patients with periodontitis, discusses the potential mechanisms of diabetic bone loss. Most studies have focused on the mechanism of the effect of diabetes on periodontitis, but there have been few studies on the mechanism of the effect of periodontitis on diabetes. Remission of periodontal infection can improve local inflammation levels, among which the key factors involved are IL-6 and TNF-α. Whether there are other important factors involved in this process remains to be explored.

Poor glycemic control has adverse effects on alveolar bone quality and quality in diabetic patients, and it is also an important parameter to judge the long-term success of implants in diabetic patients. Diabetes leads to the imbalance in oral bone metabolism, increased osteoclast activity, and decreased bone repair, which accelerates the absorption of alveolar bone. The interaction of AGE-RAGE can also aggravate the inflammatory response and affect bone metabolism. Therefore, to maintain the health of alveolar bone, stomatologists should emphasize the benefits of careful control of blood glucose, pointing out that hyperglycemia will have a negative impact on the success of oral treatments.

## Author Contributions

XW and JH conceived and designed the study. HW, LC, and TZ collected data. XW and CK wrote the paper. LC, TZ, CK, and JH reviewed and edited the manuscript. All authors read and approved the manuscript.

## Conflict of Interest

The authors declare that the research was conducted in the absence of any commercial or financial relationships that could be construed as a potential conflict of interest.
